# A Deep Learning Approach for Removing Multi-Source Transient Interference in Satellite Magnetic Field Measurement

**DOI:** 10.3390/s25247533

**Published:** 2025-12-11

**Authors:** Ning Li, Jindong Wang, Shanzhi Ye, Yiteng Zhang, Xiaochen Gou

**Affiliations:** 1 State Key Laboratory of Solar Activity and Space Weather, National Space Science Center, Chinese Academy of Sciences, Beijing 100190, China; lining211@mails.ucas.ac.cn (N.L.); yeshanzhi@nssc.ac.cn (S.Y.); ytzhang@nssc.ac.cn (Y.Z.); gxc@nssc.ac.cn (X.G.); 2 University of Chinese Academy of Sciences, Beijing 100049, China

**Keywords:** stray magnetic, multi-source transient interference, 1D convolutional neural network, magnetic data correction, SMILE, THEMIS

## Abstract

Magnetic field measurements are essential for space science missions but are often contaminated by transient stray fields from spacecraft subsystems such as electrical and control units. Traditional mitigation approaches—including strict magnetic cleanliness programs, deployable long booms, and dual-sensor gradient systems—suffer from inherent limitations, as cleanliness programs and long booms impose high cost and system complexity. To overcome these challenges, we propose Multi-Source Adaptive Gradiometry (MSAG), an enhanced gradiometry technique that integrates a neural network-based interference classification framework. The trained network identifies interference types and applies adaptive correction coefficients, enabling accurate multi-source disturbance correction without requiring manual segmentation of long-duration data. We validate MSAG using realistic synthetic data, generated by superimposing key transient interference types—modeled from SMILE ground tests—onto actual THEMIS satellite measurements, and test it through 220 Monte Carlo simulations. MSAG reduces the median RMSE from 0.457 nT to 0.014 nT and achieves a median correlation coefficient of 0.999994 with the ground truth. This improved accuracy could alleviate constraints on magnetic cleanliness and boom length in future missions, highlighting the advantage of MSAG over conventional methods and underscoring the potential of combining machine learning with gradiometry for high-fidelity magnetic field recovery.

## 1. Introduction

Magnetic field measurements are essential in space science missions, as they are key to understanding geophysical processes. For example, most space science missions deployed magnetometers to study the interaction between the solar wind and planetary environments by obtaining in-situ vector magnetic field data, such as the THEMIS mission [1] and Solar Wind Magnetosphere Ionosphere Link Explorer (SMILE) mission [2]. However, on spacecraft, time-varying stray magnetic fields can be generated by subsystems such as magnetorquers, reaction wheels, battery currents, solar panels, heaters, and other instruments [3]. These stray fields are difficult to distinguish from the geophysical signals of interest because they exhibit significant overlap in the frequency domain, and the amplitude of the interference may be similar to that of the variation in the space magnetic field.

To reduce the impact of stray magnetic fields in magnetometer measurements, a strict magnetic cleanliness program is required [4]. Another effective approach is to increase the distance from the spacecraft between the magnetometer and the spacecraft by installing the magnetometer at the end of a long deployable boom. For example, there is a 13 m long boom for the Voyager mission [5] and a 12 m long boom for the Kaguya mission [6]. However, long booms are costly and prone to distortion. Therefore, a boom of moderate length combined with algorithm processing has become the mainstream direction [2].

The dual-sensor gradiometry technique was implemented by mounting two magnetometers at different boom locations to remove stray magnetic fields [7,8,9,10,11]. This identifies the stray field variation by analyzing the measurements between the two magnetometers (ΔB), and can correct the stray field to obtain the ambient magnetic field. However, real-world stray fields are time-varying and have nontrivial multipole harmonics are more challenging to remove. Some statistical methods have already been developed and applied, including Principal-Component Gradiometry (PiCoG) and Independent Component Analysis method (ICA) [8,12]. The PicoG systematically identifies orthogonal components through maximum variance analysis, with subsequent estimation of coupling coefficients to remove stray magnetic field. The ICA method is based on the assumption that stray signals are independent and non-Gaussian distributed, and estimates the ambient magnetic field through the calculation of a mixing matrix. However, these methods are generally effective only for a limited number of interference types, and their performance degrades when multiple complex or overlapping disturbances are present.

For satellites without a boom or with a short boom, a series of noise cancellation methods have been developed, such as blind source separation method and wavelet analysis method. However, for scientific data, the boom and the gradiometry method have higher accuracy [13,14,15]. The advantage of the gradiometry approach results from applying appropriate correction coefficients for interference sources to obtain the environmental magnetic field. The difficulty is that the stray field is caused by different sources. In 1974, Ness’s use of universal correction coefficients was bound to fail to eliminate multiple interferences [16].

Recent studies have also explored spacecraft-induced magnetic interference, particularly the alternating magnetic disturbances generated by reaction wheels. By combining signal decomposition with machine-learning techniques, these methods have demonstrated strong capability in isolating platform-generated AC interference from the ambient magnetic field [3]. Such results further support the use of data-driven interference mitigation in spacecraft magnetic field measurements.

The Solar Wind Magnetosphere Ionosphere Link Explorer (SMILE), a joint mission of ESA and CAS designed to investigate solar wind–magnetosphere coupling processes, employs a dual-sensor fluxgate magnetometer mounted on a 3-m boom [2,17]. During the mission’s ground magnetic noise tests, several representative types of transient interference were identified. To construct realistic validation scenarios, these interferences were mathematically modeled and superimposed on THEMIS in-situ magnetic field measurements. Building on traditional gradiometry, we propose Multi-Source Adaptive Gradiometry (MSAG), which integrates neural-network-based classification with adaptive correction to enhance interference suppression. Applied to the synthetic datasets, MSAG reduces the root mean square error (RMSE) by 96.3% relative to the outboard sensor measurements and achieves an additional 22.8% improvement over conventional gradiometry.

## 2. Methods

The traditional gradiometry for removing stray fields is mainly achieved by utilizing the measurement differences of multiple magnetometers [9]. Taking one axial direction of the magnetometer as an example, assume that the measured magnetic field value of the magnetometer is $B_{n}$, where n=1, 2 represents the label of sensors. The magnetic field measurement by the magnetometer in space can be expressed by the following formula:

(1) $$B_{n}=B_{am}+b_{in}+w_{n}$$

where $B_{am}$ is the ambient magnetic field measurement by the magnetometer, $b_{in}$ is the measured interference magnetic field. The interference fields measured by the two magnetometers are different. i=1…m, represents the type of interference magnetic field. $w_{n}$ is random normal noise terms accounting for measurement errors [14].

In the commonly used dual-sensor gradiometry method in space science missions, the magnetic field at each magnetometer can be expressed by the following formulas:

(2) $$\left\{\begin{array}{c} B_{1}=B_{am}+b_{i1}+w_{1}\;\\ B_{2}=B_{am}+b_{i2}+w_{2} \end{array}\right.$$

where 1 and 2 are the magnetometer installed near and far from the spacecraft. The interference magnetic field $b_{i1}$ of the spacecraft is much larger in magnitude than random normal noise signal w; thus, the latter’s influence is ignored when calculating the ambient magnetic field.

To obtain the ambient magnetic field from the dual-sensors configuration, a proportional coefficient $k_{i}$ is introduced [9]. The coefficient can be expressed as:

(3) $$k_{i}=\frac{B_{2}(t+d_{t})-B_{am}(t)}{B_{1}(t+d_{t})-B_{am}(t)}$$

where $B_{1}\left(t\right)$, $B_{2}\left(t\right)$, and $B_{am}\left(t\right)$ are all the time-dependent magnetic field measurements. We assume that the ambient magnetic field remains unchanged during a short time segments $d_{t}$.

From the above formulas, we can obtain:

(4) $$B_{am}=(B_{2}-k_{i}\cdot B_{1})/(1-k_{i})$$

For each type of interference in each axis, there is a suitable proportional coefficient k used to eliminate the stray magnetic field and recover the ambient magnetic field. According to the Equation (4), the proportional coefficient k can be obtained, and then the ambient magnetic field can be calculated.

### 2.1. Multi-Source Transient Magnetic Interference Cancellation

The stray magnetic fields on spacecraft platforms arise from diverse electromagnetic interference sources, including power system fluctuations, electronic component emissions, and mechanical vibrations. These interference sources typically exhibit random temporal characteristics or follow periodic frequency patterns. Consequently, multiple interference fields often coexist within the same observation window, leading to spectral overlap and combined magnetic field distortions. During the ground-based magnetic noise test of the Smile mission, concurrent interference patterns were experimentally observed. They were caused by the simultaneous presence of multiple interference sources. The presence of concurrent interferences poses challenges for traditional gradiometry in correcting magnetic field data.

Traditional gradiometry methods typically model multiple interference sources as magnetic dipoles centered at the satellite’s geometric center, applying a uniform proportional coefficient for magnetic field correction [9]. This simplification assumes that interferences propagate uniformly, which fails to account for the spatial distribution of actual sources (e.g., onboard instruments at distinct positions) and the concurrent superposition of diverse interference fields. In high-precision magnetic field detection missions, such an approach introduces systematic errors due to mismatches between the idealized dipole model and real-world multi-source interferences.

The challenge escalates with concurrent interferences of independent spatiotemporal features, as single-coefficient correction fails to resolve their combined effects. This necessitates improving traditional gradiometry for multi-source adaptive interference processing, ensuring measurement accuracy in complex environments.

As described in traditional gradiometry, interference correction coefficients can be obtained for different interference sources. The coefficient $\left(k_{i}\right)$ is defined as:

(5) $$\left\{\begin{array}{c} b_{i2}=B_{2}\left(t+d_{t}\right)-B_{am}(t)\\ b_{i1}=B_{1}\left(t+d_{t}\right)-B_{am}(t) \end{array}\right.$$

(6) $$k_{i}=\frac{b_{i2}}{b_{i1}}$$

Similar to Equation (3), in Equation (5) $d_{t}$ represents a short time interval during which it is assumed that there is no change in the ambient magnetic field $B_{am}$. $B_{1}$ and $B_{2}$ denote the magnetic fields measured by the two magnetometers, while $b_{i1}$ and $b_{i2}$ represent the magnetic field variations caused by interference source i at the two magnetometers within the time interval $d_{t}$. The correction coefficient $k_{i}$ for such an interference source can then be calculated using Equation (6).

Neural networks can be used to identify the time intervals during which different interference sources are present. As shown in Equation (5), when only a single type of interference exists in a segments, the background magnetic field can be estimated directly. However, when two types of interference occur simultaneously, the *k* coefficient for the overlapping interval can be calculated using the following formula:

(7) $$k_{ij}=\frac{b_{i2}+b_{j2}}{b_{i1}+b_{j1}}$$

Similarly, when multiple interference types are present simultaneously, the new interference coefficient can be computed using the following formula:

(8) $$k_{i\ldots n}=\frac{\sum_{i=1}^{n}b_{i2}}{\sum_{i=1}^{n}b_{i1}}$$

By applying the appropriate k values to the identified concurrent interference segments, the effects of the concurrent interference can be eliminated. Further details can be found in the experimental results in Section 3.

### 2.2. 1D-CNN for Time-Series Data

In recent years, deep learning has made significant advancements, demonstrating broad applicability across domains such as natural language processing [18], facial recognition, autonomous driving [19], and various areas of space physics [3,20]. Convolutional Neural Networks (CNNs), in particular, have shown strong capability not only in image processing but also in handling one-dimensional data like time-series and sensor measurements. For time-series tasks, CNNs benefit from their local receptive fields, which enable them to effectively capture characteristic patterns such as peaks, trends, and short-term temporal behaviors.

Since spatial magnetic field signals are inherently time-series data, 1D-CNNs can accurately extract local features from these signals, thereby supporting reliable classification and further advancing space physics research. While recurrent models such as RNNs and LSTMs are advantageous for learning long-term temporal dependencies, the classification task in this study relies more on extracting local patterns rather than modeling long-range relationships [21,22]. Therefore, a 1D-CNN offers a more suitable and efficient solution for our application.

The core layers of a 1D Convolutional Neural Network (1D-CNN) are the convolutional layers. These layers consist of multiple filters, also known as kernels, which are used to extract localized features from the input sequence.

For a time-series input $X=\left[x_{1},x_{2},\ldots ,x_{n}\right]$ and a filter $W=\left[w_{1},w_{2},\ldots ,w_{K}\right]$, the feature map $Y=\left[y_{1},y_{2},\ldots ,y_{m}\right]$ is computed through the following convolution operation:

(9) $$y_{i}=\sum_{j=1}^{K}w_{j}\cdot x_{i+j-1}$$

where *yi* represents the output of the convolution at position *i*, and *K* is the size of the filter. The filter *W* slides over the input *X* and computes the dot product at each position, thereby producing the feature map *Y*, which captures important patterns or features from the input sequence. This operation allows the CNN network to effectively learn spatially invariant patterns from time-series data.

To enable the learning of complex and non-linear data representations, activation functions are applied to the convolutional layer outputs. The Rectified Linear Unit (ReLU), widely used in 1D-CNNs, is defined as:

(10) $$\mathrm{ReLU}\left(x\right)=\max\left(0,\;x\right)$$

ReLU accelerates model training and prevents the vanishing gradient problem, thereby improving convergence rates.

Dimensionality reduction is achieved using max pooling, which selects the maximum value from a defined window of features, reducing feature map size while retaining the most significant information. For a pooling window of size P, the operation is defined as:

(11) $$y_{i}=\max\left(x_{i},x_{i+1},\ldots ,x_{i+P-1}\right)$$

This step enhances computational efficiency and reduces overfitting risks by discarding less critical details.

Finally, the fully connected layer aggregates the extracted features into a global representation for the final prediction. The layer computes its output as:

(12) $$Z=W_{\mathrm{fc}}\cdot \mathrm{flatten}\left(Y\right)+b_{\mathrm{fc}}$$

where $W_{\mathrm{fc}}$ and $b_{\mathrm{fc}}$ denote the layer’s weights and biases, respectively.

At the end of the model architecture a few fully connected layers followed by the softmax activation function, defined as:

(13) $$softmax{\left(x\right)}_{i}=\frac{e^{x_{i}}}{\sum_{j=1}^{k}e^{x_{j}}}$$

where *x* is the input vector and *K* is the number of categories. The softmax function is used in the output layer to transform the final layer’s outputs into probabilities.

These components collectively enable 1D-CNNs to extract features from time-series data and perform classification.

### 2.3. Transient Magnetic Interference Classification and Data Correction

Spatial magnetic field data can be regarded as a type of time—series data. At present, in a large number of fields, time—series classification techniques in deep learning are used to replace manual labor in performing a large number of repetitive tasks.

For satellites with magnetic uncleanliness or those with a relatively short boom length, the data detected by magnetometers often contain a large number of data segments affected by stray magnetic field interference. These different interference signals can be accurately attributed by combining the satellite’s telemetry data, and the time segments with interference can be manually identified. However, when the satellite is in orbit, there is insufficient support from telemetry data, so a large amount of manual processing is required to obtain high—quality magnetic field data.

The stray magnetic fields generated by spacecraft have the following characteristics:

(1). The same interference source has a relatively fixed change pattern;

(2). The change patterns generated by different interference sources often have different manifestations [11].

Therefore, time-series classification techniques in deep learning can be used to classify the detected affected data, thereby determining the interference source of each segment of interference data, and using the corresponding correction coefficient to eliminate this segment of data.

**Figure 1:**
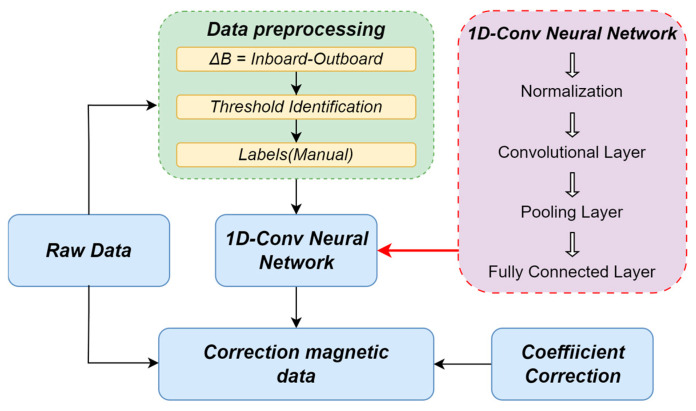
Flowchart of data processing using the MSAG method. It encompasses data preprocessing and classification through a Convolutional Neural Network (CNN). Finally, the processed data is obtained by applying the correction coefficients, which are determined according to the interference categories and calculated following Equations (6) and (8).

The Figure 1 is the flowchart of our entire algorithm:

1. First, calculate the difference of the original data to obtain ΔB.
2. To detect abrupt changes in the magnetic field signal B(t), calculate the difference ΔB between the average values of B(t) over two consecutive sliding time windows. Identify and record the data segments covered by these two windows wherever the absolute value of this difference (|ΔB|) exceeds a predefined threshold t. These identified segments correspond to periods containing potential interference.
3. The types of interference are manually identified, and corresponding classification labels are established. For each individual and composite interference, the associated correction coefficients are derived using Equations (6) and (8) and subsequently applied in Step 6 for interference elimination.
4. Input the abnormal data and classification labels into the 1D-CNN classification model for training.
5. After model normalization, convolution, pooling, and fully-connected classification, output the classification results of the interference segments.
6. Apply the correction coefficients *k* obtained in Step 3 to eliminate the corresponding interferences and thereby obtain the corrected magnetic field data.

## 3. Simulation and Experiments

**Figure 2:**
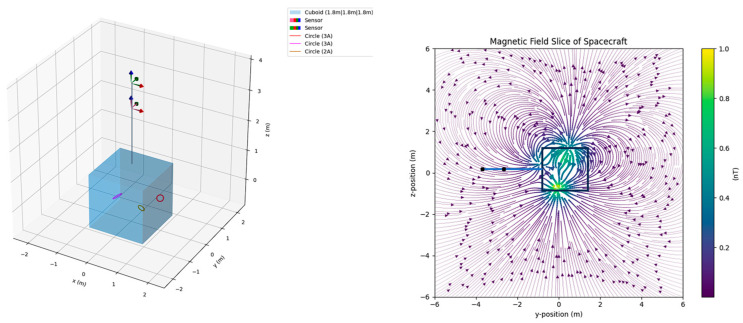
The **left** image shows the schematic of the SMILE satellite with dual magnetometers on a 3-m boom. Magnetic noise sources include thrusters and power systems positioned randomly within the spacecraft. The **right** image shows the magnetic field slice of the spacecraft.

The SMILE satellite employs a magnetically clean design, a 3-m deployable boom, and a dual-sensor fluxgate magnetometer. Ground magnetic noise tests identified several well-defined types of spacecraft-induced interference, which provide realistic scenarios for interference modeling and mitigation studies. To evaluate the effectiveness of the proposed Multi-Source Adaptive Gradiometry (MSAG) method, this chapter constructs synthetic datasets by superimposing modeled interferences onto THEMIS in-situ magnetic field measurements and carries out simulation-based magnetic field reconstruction experiments. Figure 2 shows the dual-magnetometer configuration of the SMILE mission as well as magnetic field slices of the simulated interference sources.

We use the Root Mean Square Error (RMSE) and the Pearson correlation coefficient (r) to quantitatively evaluate the performance of our method. These metrics are computed by comparing the corrected magnetic field signal $x_{i}$ with the truth reference signal $y_{i}$ over a total of N sample points.

(14) $$RMSE=\sqrt{\frac{1}{N}\sum_{0}^{N-1}{\left(x_{i}-y_{i}\right)}^{2}}$$

RMSE reflects the average magnitude of the error between the corrected and true signals. A lower RMSE indicates better correction accuracy.

(15) $$\rho =\frac{\sum_{0}^{N-1}\left(x_{i}-\bar{x}\right)\left(y_{i}-\bar{y}\right)}{\sqrt{\sum_{0}^{N-1}{\left(x_{i}-\bar{x}\right)}^{2}}\sqrt{\sum_{0}^{N-1}{\left(y_{i}-\bar{y}\right)}^{2}}}$$

The correlation coefficient ρ measures the similarity between the corrected and true signals, ranging from −1 to 1. A value of ρ = 1 indicates perfect positive correlation, and in our case, a value close to 1 signifies that the corrected signal closely follows the true signals.

### 3.1. Data Preparation and Interference Modeling

In this study, we constructed a test dataset consisting of in-situ magnetic field measurements from the THEMIS mission, onto which simulated operational interferences from the electric thruster, heater, and power amplifier systems were superimposed. The Themis mission was launched in February 2007 and operates in Earth orbit similar to that of the SMILE satellite [4,23]. The reason why these three kinds of interference data are chosen is that we found from the data in the ground tests that these interferences occur most frequently during the testing process. Moreover, these types of interference are common on other spacecraft platforms [8,11]. Additionally, a random noise with a peak-to-peak amplitude of 0.02 nT was introduced to both probes to simulate the magnetometer’s background noise.

Table 1 summarizes the three most common types of interference identified: (1) Step-like interference, (2) Spike-like interference, (3) Combined-step interference. The powering on and off of the payload also resembles composite step interference. Moreover, there are cases where two types of interference coexist. To simulate these three types of interference, we described their shapes with mathematical formulas and formulated them as follows:

(16) $$\;\left\{\begin{array}{c} I_{step}\left(t\right)=A\cdot [u\left(t-t_{0}\right)-u\left(t-t_{0}+∆t\right)]\\ I_{spike}\left(t\right)=A\cdot \delta \left(t-t_{0}\right)\\ I_{combined-step}\left(t\right)=\sum_{k=1}^{n}A_{k}\cdot [u\left(t-t_{k0}\right)-u\left(t-t_{k0}+∆t_{k}\right)] \end{array}\right.$$

Equation (16) provides a general modeling framework for different types of interference currents, where u(·) is the Heaviside step function and *δ*(·) is the Dirac delta function. The amplitude *A* (or $A_{k}$)represents the strength of the interference and varies with different sources. ∆t (for $I_{step}\left(t\right)$) and $∆t_{k}$ (for $I_{combined-step}\left(t\right)$) denote adjustable durations of the step components. For a given interference source, the duration of each occurrence is randomly selected within a specified upper and lower bound. $t_{0}$ (or $t_{k0}$) is the step start time.

These interferences are operationally triggered, and we assume their occurrence is random. To better reflect realistic in-orbit conditions, their waveforms are fine-tuned with an amplitude variation ranging from −5% to +5% relative to nominal values. This modification enhances the realism of the simulated interference signals while preserving their characteristic patterns.

The SMILE satellite is equipped with a 3-m-long boom that carries a dual-sensor mounted at different distances from the satellite body. Our simulation follows the same configuration. To model onboard magnetic interferences, we used Magpylib [24], an open-source Python 3.11 package for calculating static magnetic fields generated by magnets, current loops, and other sources. We randomly placed simulated interference sources inside a satellite body using Magpylib. By adjusting parameters such as the current magnitude and the loop diameter, we ensured that the resulting magnetic field amplitudes at the inner and outer sensors matched those observed during ground testing.

**Figure 3:**
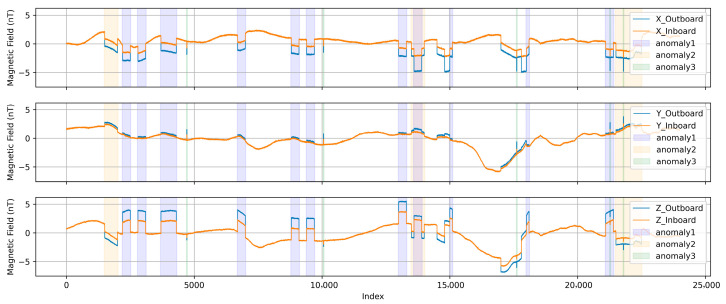
Segment of THEMIS-A magnetic field data from 10 March 2025, with added synthetic interference. Shaded areas in different colors denote different interference types.

Figure 3 shows a segment of THEMIS A magnetic field data on 10 March 2025, with an interference signal. The THEMIS data used here has been preprocessed by subtracting the mean value to remove the background DC component, focusing solely on AC variations.

### 3.2. Interference Classification with CNN

We applied the transient interference identification method described in Section 2 to detect interference segments, which were subsequently labeled to construct a dedicated dataset. This dataset was split into training and test sets for model development and evaluation. A convolutional neural network (CNN) was trained using the training set. The network adopts a multi-stage architecture optimized for effective feature extraction and generalization. The model begins with an input layer, followed by a min-max normalization layer to scale the input data. A convolutional layer with dropout (Conv-drop) is used to extract local features while preventing overfitting. This is followed by a max-pooling layer for spatial downsampling. The resulting feature maps are flattened through a Flatten-drop layer, which also applies dropout regularization. Two fully connected (FC) layers are used to refine the learned representations, leading to the final output layer for classification. This architecture achieves a balance between feature learning, dimensionality reduction, and regularization, resulting in excellent performance—achieving over 99% classification accuracy on value sets.

**Figure 4:**
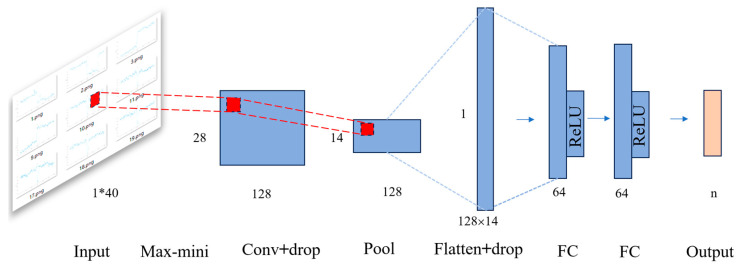
Architecture of the convolutional neural network for magnetic data classification, including normalization, convolutional, pooling, dropout, and fully connected layers.

The overall structure of the classification network is illustrated in Figure 4. We conducted ablation experiments with different kernel sizes and channel numbers, based on which we ultimately selected 128 channels and a kernel size of 13. The network was implemented in Python using the PyTorch framework and trained on an NVIDIA GeForce RTX 4060 Ti GPU for 500 epochs.

**Figure 5:**
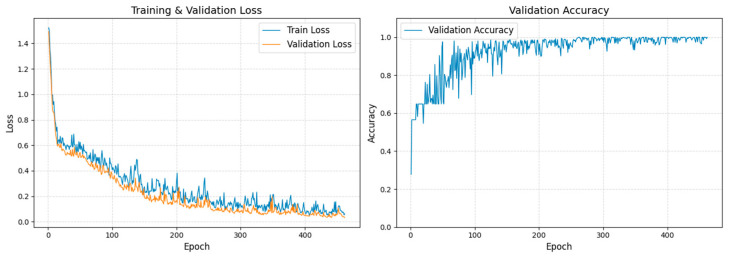
Training and validation performance of the CNN model. (**Left**) Loss curves showing stable convergence. (**Right**) Validation accuracy approaching 100%, indicating excellent generalization.

During the training process, the validation loss gradually decreased and eventually converged to a low level, while the validation accuracy steadily increased and ultimately reached 100%, demonstrating the model’s excellent generalization capability. The smooth trend observed in the loss curve indicates that batch normalization effectively mitigated gradient fluctuations, promoting stable and reliable convergence. These results validate the effectiveness of the network design and training strategy. The overall training dynamics, including loss and accuracy evolution, are depicted in Figure 5.

The results in Figure 5 clearly demonstrate that the 1D-CNN can reliably distinguish different interference types, providing accurate classifications for subsequent data correction.

**Figure 6:**
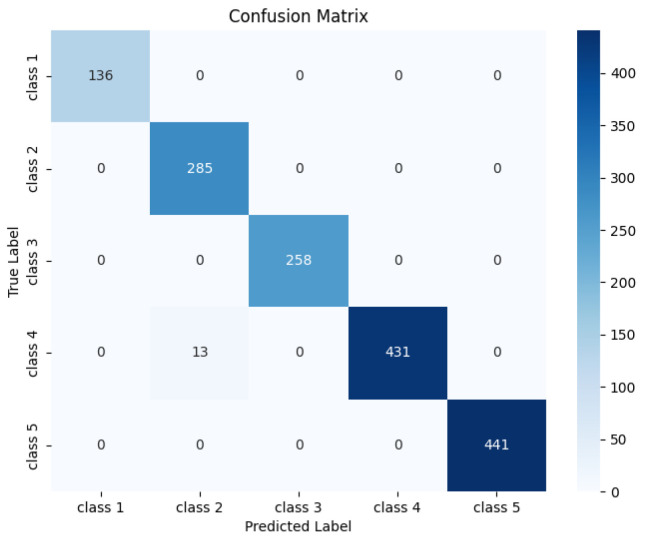
Confusion Matrix of the 1D-CNN. Rows show true label, and columns show predicted label. Diagonal values indicate correctly classified samples. Off-diagonal values show misclassifications.

Figure 6 presents the confusion matrix of the proposed classifier, and Table 2 summarizes the corresponding precision, recall, and F1-scores for each interference class. Although three types of interference sources were introduced in the experiment, as illustrated by the colored backgrounds in Figure 3, the network was trained to categorize them into five classes to reflect their distinct physical characteristics. Class 1 corresponds to the spike-like interference from Anomaly 3, while Classes 2–5 are associated with the start and end segments of the step-like interferences produced by Anomalies 1 and 2. Separating these interferences into start and end transitions allows each interference category to be linked to the underlying on/off behavior of the interference sources.

Under this classification scheme, the active interval of each interference source—identified by its detected start and end edges—can be accurately determined. This ensures that the magnetic-field data can be corrected using the appropriate compensation coefficients, independent of the duration of each interference event.

As demonstrated by the confusion matrix in Figure 6 and the quantitative metrics in Table 2, the CNN achieves good performance in the five-class classification task, confirming that the network can effectively recognize and separate the different types of interference for subsequent data correction.

### 3.3. Magnetic Field Correction

**Figure 7:**
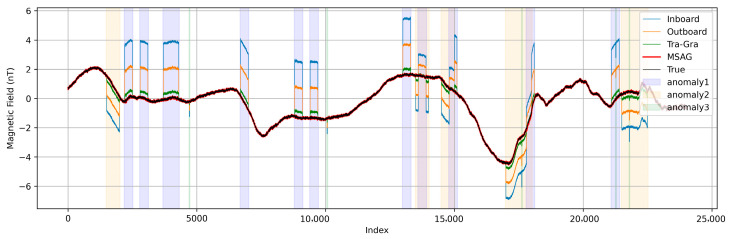
THEMIS magnetic field segment with added interferences. Inboard magnetometer measurements are shown in blue, outboard magnetometer measurements in orange, MSAG-corrected data in red, traditional gradiometry-corrected data in green, and the reference THEMIS data without interferences in black.

Based on the interference classifications produced by the neural network in Section 3.2, the MSAG method corrects the magnetic field by applying the tailored coefficients for single-type and combined transient interferences (Equations (6) and (8)) through Equation (4). For comparison, the results obtained using the traditional gradiometry(Tra-Gra) method are also shown in Figure 7. Here, only the Z-axis correction results are presented, while the same approach is applicable to the other axes.

The traditional-gradiometry method models interference sources as equivalent magnetic dipoles located at the satellite’s geometric center, applying geometric correction factors to suppress multiple interference components. As shown in Figure 7, this approach (green trace) provides a notable improvement over the outboard-sensor measurement but still exhibits residual deviations compared to the truth signal (black trace). In contrast, the MSAG algorithm (red trace) achieves a near-perfect match with the ground truth by leveraging deep learning to model and suppress complex, transient interference. These results highlight the superiority of data-driven approaches over traditional geometric correction methods in mitigating stray magnetic interference.

**Figure 8:**
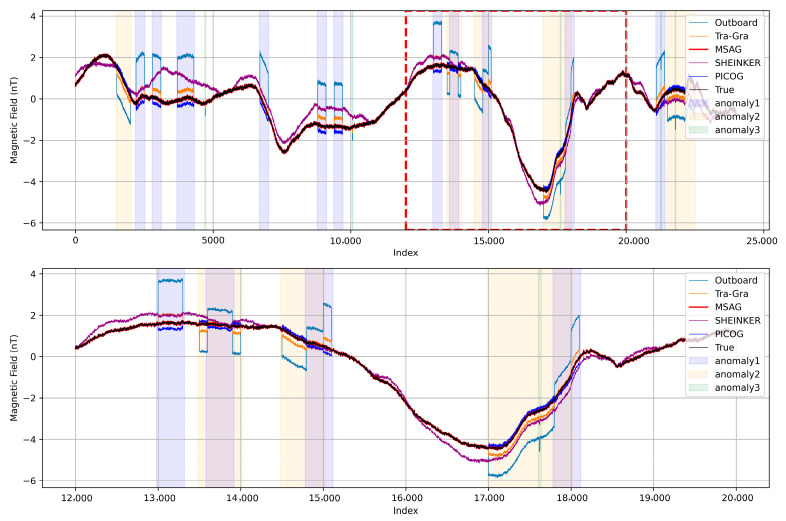
Comparison of the proposed MSAG method with selected algorithms, including Tra-Gra, SHEINKER, and PICOG. Shaded regions denote time intervals affected by interference. The red dashed box highlights a specific segment, which is magnified below for a more detailed comparison.

In addition to the traditional gradiometry method, numerous other algorithms have been developed for mitigating stray magnetic interferences. MagPrime is an open-source library that encompasses most existing magnetic interference removal techniques [15]. To further evaluate the performance of the MSAG method, we selected the SHEINKER and PICOG algorithms from this library for comparison. Both SHEINKER and PICOG are suitable for interference mitigation in a dual-sensor configuration. Other methods, such as REAM and WAIC-UP, are more effective for interferences with pronounced frequency-domain characteristics and are therefore not included in this comparison [15]. Additionally, ICA is only capable of removing three specific types of interference in a dual-sensor setup and is not applicable in this context [12]. Figure 8 presents the processing results of the MSAG method alongside these selected algorithms.

To ensure fairness in the comparison among Tra-Gra, SHEINKER, and PICOG, the parameters for each method were configured according to their theoretical assumptions and practical applicability to the present dataset. For the Tra-Gra method, the interference source is modeled as a magnetic dipole located at the spacecraft’s geometric center; thus, the correction factor is determined solely by the geometric relationship and is computed as $k={\left(r_{1}/r_{2}\right)}^{3}$, requiring no additional tuning. The SHEINKER method assumes that both the ambient magnetic field and the interference field have zero mean. In this study, the DC component of the ambient field was removed during preprocessing, and the magprime implementation employs a sliding-average detrending step to satisfy the zero-mean interference assumption. However, because the transient interference exhibits random onset times and varying durations, the selection of the detrending window is challenging: a window that is too short risks incorporating interference into the trend estimate, whereas an overly long window may distort the ambient field during non-interference intervals. A window length of 2000 samples was empirically identified as an effective compromise under the present experimental conditions. For the PICOG method, the correction order is the key configurable parameter. Several candidate orders were evaluated, and the second-order configuration provided the best performance for the characteristics of the dataset used in this work. These parameter choices ensure that each method operates under conditions consistent with its theoretical requirements and practical strengths.

The transient interference examined in this study exhibits random durations and variable temporal characteristics, which increases the difficulty of interference suppression. As shown in Figure 8, the detrending and subsequent trend re-addition in the SHEINKER method may introduce slight interference-like components during non-interference intervals, such as in the range of samples 5000–10,000. Moreover, since the method is formulated for scenarios involving only a single interference component, its performance may degrade when multiple interference types coexist. For the PICOG method, although the second-order configuration was found to yield the most favorable results among the tested settings, certain residual components remain visible in the processed data.

Overall, the proposed MSAG method enhances spacecraft magnetic field data by integrating gradiometry-based stray field removal with deep learning-based interference classification. Through accurate identification of interference types and the application of tailored correction coefficients, MSAG effectively reduces RMSE and improves data fidelity. These results highlight the potential of combining machine learning with traditional signal processing techniques to advance the quality and reliability of spaceborne magnetic field measurements.

### 3.4. Monte Carlo Simulation Results and Analysis

To quantitatively assess the performance of the algorithm, 220 simulations were conducted using the open-source MagPrime package, with each trial incorporating three distinct interference sources embedded in the satellite structure. Root Mean Square Error (RMSE) analysis indicates notable improvements achieved by the MSAG algorithm. The method provides accurate correction during interference events while maintaining data integrity during intervals without interference.

**Figure 9:**
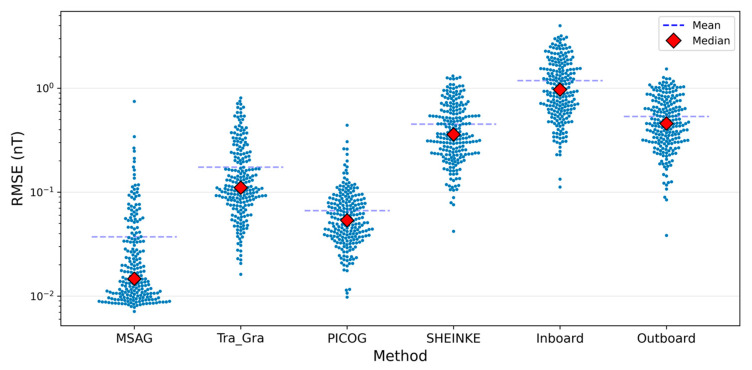
RMSE of the Z-axis magnetic field after correction by different algorithms, and the original outboard and inboard sensor measurements. MSAG exhibits smaller median and mean RMSE values compared with the other methods in this study.

Comparative evaluation using the MagPrime library further indicates that MSAG offers more reliable correction under the multi-source transient interference conditions considered in this study. As shown in Figure 8, all tested algorithms mitigate part of the distortion relative to the unprocessed outboard data; however, residual artifacts can still be observed in several cases. In contrast, MSAG is specifically designed to address multi-source transient interference, which results in more stable performance. The RMSE statistics in Figure 9 corroborate this observation, with MSAG exhibiting a smaller median error compared to the other algorithms.

Each of the 220 Monte Carlo simulations involved 300,000 data points. Figure 8 presents representative segments of the simulated signals, highlighting intervals where multiple interferences occur simultaneously. Figure 9 shows the RMSE distributions computed over the full 300,000-point simulations for each algorithm, providing a comprehensive view of performance across the entire dataset; correspondingly, the median statistics summarized in Table 3 are also derived from these complete simulations, collectively demonstrating the notable noise suppression capabilities of the MSAG method. After correction, the median RMSE for outboard sensor measurements is reduced from 0.457 nT to 0.014 nT—an order-of-magnitude improvement that underscores the method’s precision in isolating transient disturbances from the ambient magnetic field. In addition to RMSE reduction, MSAG achieves the highest correlation with the ground truth signal among all compared algorithms, with a median correlation coefficient of 0.999994, indicating near-perfect agreement.

As demonstrated in Section 3, the proposed method effectively identifies satellite-induced dynamic interference fields directly from raw magnetic field data and classifies the corresponding interference sources. Based on the classification results, appropriate correction coefficients are applied to correct the magnetic field. Compared with the traditional dual-sensor method, the corrected magnetic field achieved by this approach shows better performance, with the RMSE improvement rising from 73.5% to 96.3%—an increase of over 22.8%.

To make the effects inside and outside interference windows explicit, Table 4 reports RMSE and correlation coefficients for the two regimes separately. MSAG maintains consistently low RMSE in both regimes and very high correlation with the reference signal. Tra-Gra and PICOG show relatively small errors outside interference windows but experience larger deviations when interference is present. SHEINKER exhibits more pronounced differences between the two regimes, showing notably higher RMSE and reduced correlation. This behavior is consistent with the method’s underlying assumptions: it requires the interference field to be zero-mean, and it is designed primarily for a single interference source. These separate evaluations help clarify how each algorithm responds to interference and how much baseline distortion, if any, is introduced outside the interference windows.

A key strength of this method lies in the integration of a neural network model, which, after learning the characteristics of various interference patterns, can rapidly recognize and accurately correct similar types of disturbances. This not only enhances correction precision but also enables the handling of multiple concurrent interference sources—an area where conventional methods often struggle. Furthermore, this automation significantly improves processing efficiency by allowing the trained network to process long-duration data segments directly, without requiring manual segmentation or intervention, thereby reducing labor-intensive preprocessing.

## 4. Conclusions and Discussion

In this study, we modeled three typical transient magnetic disturbances commonly observed on spacecraft—step-like, spike-like, and combined step disturbances—identified during the SMILE mission’s ground magnetic noise tests and attributed to subsystems such as electric thrusters, heaters, and power amplifiers. Mathematical models were established for each type, and simulations were conducted using the Magpylib library [24]. The modeled disturbances were superimposed onto real THEMIS satellite data to generate synthetic datasets for realistic validation.

A 1D-CNN was applied to classify these disturbances in the synthetic data. The dataset consisted of 300,000 points covering multiple combinations of the three interference types and was divided into training, validation, and test sets. The model was trained on the training set and validated accordingly, achieving accurate recognition of both interference type and active temporal duration. Type-specific correction coefficients were then applied, yielding highly accurate corrections. Compared to traditional methods, the data-driven MSAG approach dynamically adapts to the interference type, achieving significantly more precise adjustments. In 220 Monte Carlo simulations, MSAG reduced the median RMSE from 0.110592 nT to 0.014699 nT and improved the median correlation coefficient from 0.999677 to 0.999994.

This study focuses specifically on transient magnetic disturbances; persistent AC interference, such as that generated by reaction wheels, falls outside its scope. Comparisons with several algorithms from the open-source MagPrime library [15] show that some methods introduce minor corrections even in undisturbed segments, potentially distorting the baseline magnetic field. The MSAG method inherently avoids this issue by precisely identifying active interference periods, maintaining the original baseline data. A limitation of the supervised 1D-CNN is its reliance on comprehensive training data; interference types absent from the training set may reduce correction accuracy, highlighting the importance of datasets that cover the full range of relevant disturbances. To address unknown interference in future work, the framework could be enhanced with a “probability threshold mechanism.” In this approach, if MSAG determines that a sample does not correspond to any known interference type, the data would not be processed immediately but instead be marked for subsequent detailed manual analysis.

Overall, these results demonstrate the strong potential of neural network-based, data-driven methods for correcting transient spacecraft-induced magnetic interference. The MSAG method is expected to be applicable to the magnetic field measurements of the upcoming SMILE mission. Future work should explore more robust model architectures and semi-supervised or unsupervised learning strategies to enhance generalization and reliability.

## Figures and Tables

**Table 1:** Simulated characteristics of three major onboard magnetic interference sources.

Noise Source	Typical Cause	Waveform Characteristics	Amplitude Variation
Electric Thruster	Propulsion system	Combined-step like	Repeated pattern ±5% variation per occurrence
Heater	Thermal control subsystem	Spike-like	
Power Amplifier	Communication system	Step-like	

**Table 2:** Precision, Recall, and F1-Scores for Each Interference Class.

Class	Precision	Recall	F1-Score
Class1	1	1	1
Class2	0.9564	1	0.9777
Class3	1	1	1
Class4	1	0.9707	0.9851
Class5	1	1	1

**Table 3:** Correction Methods Performance (Median Statistics of 220 Experiments).

Method	RMSE (nT)	Correlation Coefficient	RMSE Reduction Compared to Outboard Sensor
MSAG	0.014699	0.999994	96.3%
Tra-Gra	0.110592	0.999677	73.5%
SHEINKER	0.359734	0.997356	17.8%
PICOG	0.053620	0.999923	88.5%
Outboard Sensor	0.457191	0.994434	——
Inboard Sensor	0.969578	0.976248	——

**Table 4:** Correction Methods Performance: Interference/Non-interference (Median Statistics of 220 Experiments).

Method	RMSE (nT)		Correlation Coefficient	
	Interference	Non-Interference	Interference	Non-Interference
MSAG	0.021106	0.005779	0.999986	0.999999
Tra-Gra	0.229096	0.012370	0.999189	0.999995
SHEINKER	0.515717	0.297467	0.996018	0.998219
PICOG	0.112115	0.003483	0.999806	0.999999

## Data Availability

The data that support the findings of this study are available upon reasonable request from the authors. The THEMIS satellite data used in this study are publicly available from the THEMIS data repository at https://themis.ssl.berkeley.edu/data/themis/tha/l2/fgm/ (accessed on 10 March 2025).
